# Controlling the Electronic Structures and Properties of in-Plane Transition-Metal Dichalcogenides Quantum Wells

**DOI:** 10.1038/srep17578

**Published:** 2015-11-30

**Authors:** Wei Wei, Ying Dai, Chengwang Niu, Baibiao Huang

**Affiliations:** 1 School of Physics, State Key Laboratory of Crystal Materials, Shandong University, Jinan 250100, China; 2 Peter Grünberg Institut and Institute for Advanced Simulation, Forschungszentrum Jülich and JARA, 52425 Jülich, Germany

## Abstract

In-plane transition-metal dichalcogenides (TMDs) quantum wells have been studied on the basis of first-principles density functional calculations to reveal how to control the electronic structures and the properties. In collection of quantum confinement, strain and intrinsic electric field, TMD quantum wells offer a diverse of exciting new physics. The band gap can be continuously reduced ascribed to the potential drop over the embedded TMD and the strain substantially affects the band gap nature. The true type-II alignment forms due to the coherent lattice and strong interface coupling suggesting the effective separation and collection of excitons. Interestingly, two-dimensional quantum wells of in-plane TMD can enrich the photoluminescence properties of TMD materials. The intrinsic electric polarization enhances the spin-orbital coupling and demonstrates the possibility to achieve topological insulator state and valleytronics in TMD quantum wells. In-plane TMD quantum wells have opened up new possibilities of applications in next-generation devices at nanoscale.

Interest in achieving atomic thin two-dimensional materials has increased since the successful access of graphene^1^. Hereinto, a class of *quasi*-two-dimensional transition-metal dichalcogenides (TMDs, i.e. MX_2_ with M being Mo or W, while X being S, Se and Te) has been spurring an intensive research activity due to their exotic physical and optical properties^2^,^3^,^4^. A diverse range of possibilities of applications in next-generation flexible nanodevices has been unraveled in TMDs^5^,^6^,^7^,^8^. MoS_2_ monolayer, for example, is gaining in importance as promising channel material for field-effect transistors with the on/off ratio exceeding 10^8^9^.

In 2014, in-plane TMD heterostructures have been realized experimentally^10^,^11^,^12^,^13^,^14^. As a central concept in modern materials science and technology^15^,^16^, with the formation of one-dimensional interfaces, in-plane TMD heterostructures visualize the interfacing of two-dimensional semiconductor materials. It is highly interesting that the perfect match in the in-plane monolayer heterostructures of two constituent TMDs marks the ultimate thickness limit for junctions between semiconducting materials up to now^14^. In contrast to the vertically stacked TMD heterobilayers^17^,^18^,^19^,^20^, which are held together by weak Van der Waals forces, atoms of constituent TMDs located at the heterointerfaces are linked by covalent bonds, due to which the in-plane TMD heterostructures ensure the epitaxial quality and thus boost the optical and electronic performance of the heterostructures. In in-plane heterostructures, distinct difference in electronic and optical features between the two joined TMDs gives rise to the importance and interest of such heterostructures. As an example, in-plane heterostructures of TMDs demonstrate type-II band alignment with electrons and holes confined at opposite sides of the interface, enabling efficient separation and collection of excitons^12^,^13^. In addition, atomically sharp interfaces and coherent lattice could generate strongly localized photoluminescence enhancement and intrinsic p-n junctions in in-plane TMD heterostructures^11^,^12^,^13^. The formation of in-plane TMD heterostructures has opened up new unprecedented opportunities to engineer two-dimensional heteromaterials with exciting new physics and applications in electronics, optoelectronics, photovoltaics, as well as photocatalysts.

In this article, we extend the concept of TMD interfaces to TMD quantum wells in light of the advances in experiments. In principle, the quantum confinement effects will add to the properties and offer new phenomenon in quantum wells. In in-plane quantum wells of TMDs, in particular, the intrinsic polarization filed and the piezoelectric polarization induced by strain will also play an important role to collectively control the electronic structures. In conjunction with other ways, for example, doping and applying external electric filed, exciting properties concealed in diverse TMD quantum wells are expected to be unraveled. Aiming at revealing how these factors and the thickness of embedded TMD will affect the electronic properties, the first-principles density functional theory (DFT) calculations have been performed and the results indicate that TMD quantum wells hold great promise in a wide range of applications.

## Results and Discussion

In comparison to the bulk counterparts, indirect-direct band gap crossover occurs in TMD monolayers due to the absence of inversion symmetry^21^. The band structures of TMD monolayers share similarities (see Figure S1, supplementary information), with the direct band gap located at the *K*-point of two-dimensional hexagonal Brillouin zone. In TMD monolayers, edge states at *Γ*- and *K*-point exhibit distinct behavior with respect to the interlayer interaction, strain and external electric filed due to the different wave function characters. This has been confirmed, for example, by the indirect-direct band gap transition in MoS_2_/WS_2_ heterobilayers due to the change of valence band maximum (VBM) position at the *Γ*-point as a function of the interlayer distance^22^,^23^. As discussed later, such a similar phenomenon can also be found in in-plane TMD quantum wells; the position of band edge will be substantially affected by the intrinsic electric polarization, strain induced by lattice mismatch as well as the thickness of embedded TMD. In TMD monolayers, giant spin-splitting of VBM at the *K*-point caused by spin-orbital coupling occurs, as a consequence of the loss of inversion symmetry in the monolayer case^24^,^25^. In in-plane quantum wells of TMDs, as discussed later, such a spin degeneracy of VBM is dramatically broken by intrinsic electric polarization. Table 1 summarizes the lattice constant *a* of TMD monolayers; and our results at PBE level of theory are in excellent agreement with previous theoretical results^26^.

In addition to the quantum confinement effects, the lattice mismatch of constituent TMDs gives rise to strain in the interface region (or piezoelectric polarization), further affecting the electronic features of TMD quantum wells. It implies that

1. In the case of large compressive strain (see the lattice constant summarized in Table 1), i.e., in WS_2_/MoTe_2_/WS_2_ and WSe_2_/MoTe_2_/WSe_2_ quantum wells, band structures demonstrate an indirect band gap, with VBM at the *Γ*-point significantly higher than that at the k-point on two-thirds way from the *Γ*- to *K*-point (referred to as *A*-point), see Figures S2(a,b), supplementary information.
2. In the case of large tensile strain, i.e., in WTe_2_/MoS_2_/WTe_2_ and WTe_2_/MoSe_2_/WTe_2_ quantum wells, the band structures reveal metallic characters, see Figures S2(c,d), supplementary information.
3. In the absence of strain, i.e., in MoS_2_/WS_2_/MoS_2_ and WS_2_/MoS_2_/WS_2_ quantum wells, band structures indicate direct band gap; both the conduction band minimum (CBM) and VBM locate at the *A*-point, see Figures S3(a,c), supplementary information. The energy difference between VBM at *Γ*- and *A*-point is rather small. As discussed later, nevertheless, increasing the thickness of embedded WS_2_ or MoS_2_ can shift the position of VBM at the *Γ*-point upward and gives rise to a direct-indirect band gap crossover in the MoS_2_/WS_2_/MoS_2_ and WS_2_/MoS_2_/WS_2_ quantum wells. This is caused by the change in intrinsic electric polarization.
4. In the presence of relatively small compressive strain, i.e., in MoS_2_/WSe_2_/MoS_2_ and WS_2_/MoSe_2_/WS_2_ quantum wells, band structures show direct band gap located at the *A*-point. The energy difference between VBM at *Γ*- and *A*-point is relatively large, see Figures S3(b,d), supplementary information.
5. The electronic structures of TMD quantum wells could be engineered by adjusting the strain.

It should be pointed out that TMD quantum wells with large compressive strain are probably unrealistic to realize in experiments due to the huge asymmetry in mechanical instability in two-dimensional structures (see below). However, we present the results in this work as extreme cases to show how the strain adjust the electronic properties. In other words, applying appropriate external strain (from compressive to tensile) to a certain TMD quantum well can also engineer the electronic properties.

**Figure 1:**
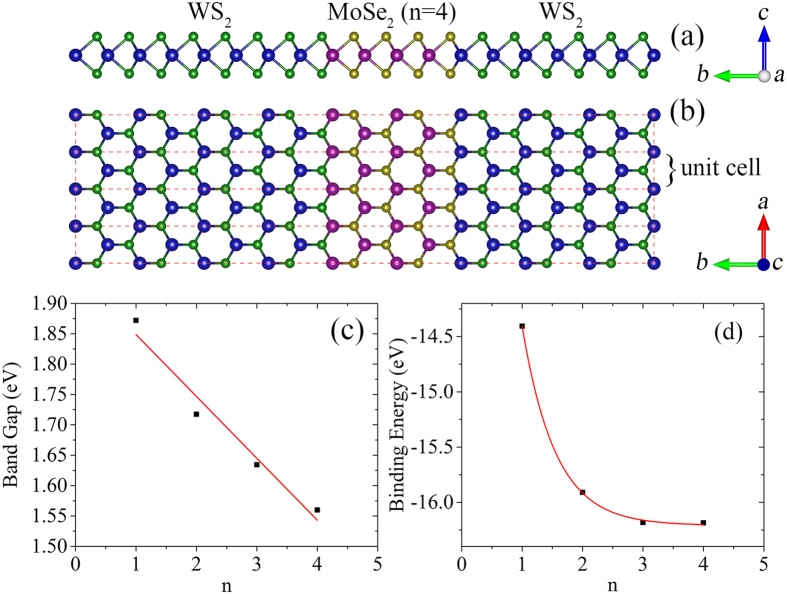
Atomic model representing the TMD quantum wells, the small spheres are non-metal atoms (S, Se and Te), while big spheres are metal atoms (Mo and W); (a) side and (b) top views of WS_2_/MoSe_2_/WS_2_ quantum well with the thickness of MoSe_2_ n being 4 (n corresponds to the number of MoSe_2_ units in the unit cell of quantum well); in (b) the rectangle represent a unit cell of the quantum well. The (**c**) band gap and (**d**) binding energy of WS_2_/MoSe_2_/WS_2_ quantum well as a function of the MoSe_2_ thickness. The binding energy is calculated by subtracting the total energies of WS_2_ and MoSe_2_ from the total energy of the WS_2_/MoSe_2_/WS_2_ quantum well, defined as *E*_b_ = *E*(WS_2_/MoSe_2_/WS_2_)-*E*(WS_2_)-*E*(MoSe_2_).

**Figure 2:**
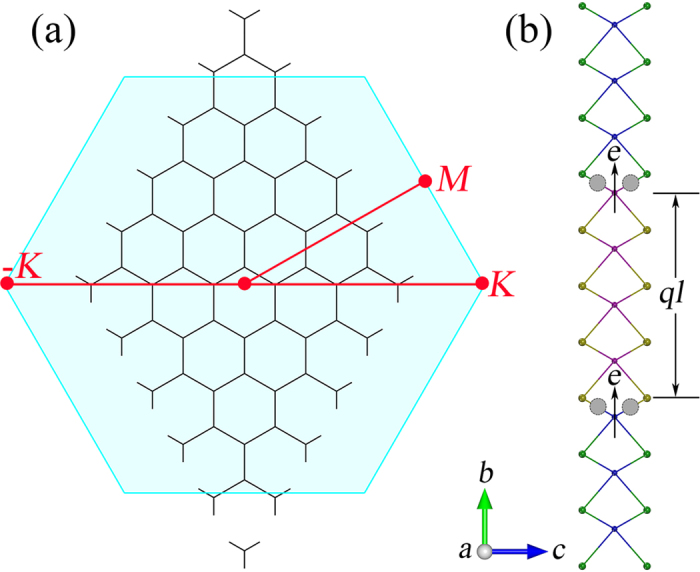
(**a**) Two-dimensional hexagonal Brillouin zone of the TMD honeycomb lattice; the *K*-point is folded at the *A*-point of rectangular Brillouin zone. (**b**) Polarization field formed in TMD quantum wells. In (**b**), the arrows show the direction of charge transfer; and shadow regions indicate covalent bonding. The dipole moment is demonstrated by an equation of *ql*.

In this work, we constructed a rectangular unit cell for the calculations, as shown in Fig. 1(a,b). In this model, dimension in *a* direction is infinite due to the periodic bound condition, while in *b* direction the length is around 70 Å. As a consequence, the band structures should be calculated in a two-dimensional rectangular Brillouin zone; however, we confirm that the band dispersion relation normal to the zigzag interfaces completely reflects the decisive characters of the band structures and we show the band structures in *Γ*-*X* direction. Although the experiments suggested in-plane TMD heterointerfaces in triangular shape with three-fold symmetry, interfacing is along the zigzag direction^10^,^11^,^12^,^13^,^14^ and this asymmetric nature is not necessary to consider in our calculations. It should be pointed out that the zigzag direction of interfacing in TMD quantum wells corresponds to the *Γ*-*K* direction of the two-dimensional hexagonal Brillouin zone, as indicated in Fig. 2(a). In consideration of band folding, the *K*-point of the hexagonal Brillouin zone is folded to the *A*-point of the rectangular Brillouin zone. In other words, that is why the VBM at *Γ*- and *A*-point manifests strong sensitivity to the interaction of constituent TMDs, as found in TMD heterobilayers^22^,^23^,^27^.

In MoS_2_/WS_2_/MoS_2_, WS_2_/MoS_2_/WS_2_ and MoS_2_/WSe_2_/MoS_2_ quantum wells, VBM and CBM wave functions distribute at opposite constituents, as shown in Figures S4(a–f), supplementary information. It indicates, in particular, the formation of type-II alignment. In low-dimensional structures, many-body effects are highlighted with the appearance of lowest-energy bound excitons, assigned to which the large binding energy^28^,^29^, the excitonic effects dominate the optical absorption properties. In in-plane quantum wells of TMDs, electrons and holes are confined at opposite constituent TMDs, facilitating the separation and collection of excitons. In particular, a type-II alignment accounts for the charge-transfer excitonic effects suggesting the possibility to realize the Bose-Einstein condensation^30^,^31^, as indicated theoretically in hydrogenated graphene^32^,^33^. Although the vertical TMD heterobilayers have also been considered to be type-II alignment^34^,^35^, a simple superposition of the optical absorption of constituent TMDs is responsible for the total spectrum due to the large interlayer distance^35^. As a consequence, true type-II alignment is established in in-plane TMD quantum wells ascribed to the coherent lattice and strong interface electronic coupling, and the monolayer-like optical properties can be expected. The graphene/TMD heterobilayers are suggested as a potential candidate in constructing the Schottky barrier solar cells^36^; however, the coupling between TMD and graphene is quite weak^37^,^38^,^39^. As discussed later, an effective band bending could be established in in-plane TMD quantum wells due to the charge transfer across interfaces. In respect to the photocatalytic water splitting of TMD^40^, formation of type-II alignment reduces the overlap of wave functions and enables effective separation of electrons and holes, thus improving the energy conversion efficiency. In essence, TMD quantum wells conceal the yet untapped potential as building blocks in applications in electronics and optoelectronics.

In the case of WS_2_/MoSe_2_/WS_2_ quantum well, both the VBM and CBM are distributed on MoSe_2_, see Figure S4(g,h), supplementary information. Taking the formation of excitons and carriers confinement effects into account, WS_2_/MoSe_2_/WS_2_ quantum wells shall enrich the photoluminescence properties of TMD materials. In order to get more details, we increases the width of WS_2_ and vary the thickness of MoSe_2_ from n = 1 to 4; while n corresponds to the number of MoSe_2_ units in a unit cell of the WS_2_/MoSe_2_/WS_2_ quantum well. Although the thickness of embedded MoSe_2_ is rather small, namely, when n = 1 or 2, the results for them are presented to see the change of electronic structures as a function of the thickness and get the main trends.

**Figure 3:**
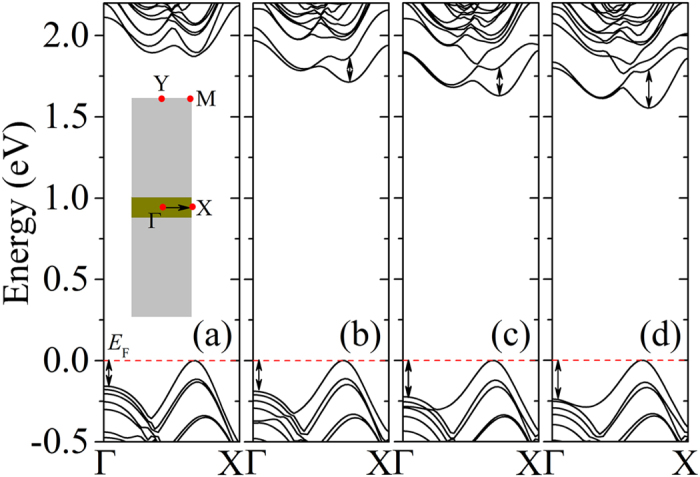
The evolution of the band structure of the WS_2_/MoSe_2_/WS_2_ quantum wells with increased MoSe_2_ thickness from (a) n = 1 to (d) n = 4. Inset in (**a**) shows the rectangular Brillouin zone of WS_2_/MoSe_2_/WS_2_ quantum wells. The arrows show the change of CBM at the *A*-point and VBM at the *Γ*-point; the dashed horizontal lines represent the Fermi level.

Figure 3 shows the evolution of band structure of WS_2_/MoSe_2_/WS_2_ quantum well with the thickness of MoSe_2_ varying from n = 1 to 4. The band gap locates at the *A*-point and almost linearly decreases with the increasing of MoSe_2_ thickness, as indicated in Fig. 1(b). In Fig. 3, the arrows represent the change of VBM position at the *Γ*-point and CBM position at the *A*-point. As MoSe_2_ thickness increases, both VBM at the *Γ*-point and CBM at the *A*-point shift downward; mini-bands tend to form at the bottom of conduction band. At the *Γ*-point, a gradual increase of an energy level can be traced in the band structures. It can be reasonably extrapolated that this energy level will further increase as the MoSe_2_ thickness increases, and a direct-indirect band gap crossover can be foreseen when the MoSe_2_ thickness is beyond a critical value. It should be pointed out that the band gap of the WS_2_/MoSe_2_/WS_2_ quantum well will probably approach to a constant value (on the basis of band alignment of WS_2_ and MoSe_2_) when the thickness of MoSe_2_ is sufficiently large. As the thickness of MoSe_2_ increases within a limitation, the electric field becomes stronger and the gap nature can be continuously tuned^41^,^42^. Figure 1(c) indicates the binding energy of forming the quantum well, defined as the energy difference between the quantum well and the constituent TMDs. The binding energies of WS_2_/MoSe_2_/WS_2_ quantum wells are fairly negative, revealing the formation of covalent bonds, and rapidly to be convergent due to the effective screening of the interface interactions as the MoSe_2_ thickness increases.

In addition to form the covalent bonds, charge transfer occurs at the one-dimensional interfaces along zigzag direction. As shown in Fig. 2(b), directions of charge transfer at interfaces are identical thus, importantly, creating an electric field over the embedded TMD in the quantum wells. The piezoelectric polarization induced by the strain also plays a role in promoting the charge transfer to give rise the electric field over the embedded TMD in the quantum wells. The charge-transfer induced electric field leads to a potential drop over the embedded TMD, and makes the electron and hole states to separate. As the thickness of embedded TMD increases, electron and hole energy becomes close and thus the band gap is significantly reduced. A similar phenomenon has also been found in GaN/InN/GaN and GaAs/Ge/GaAs quantum wells^41^,^42^. In TMD quantum wells studied in this work, quantum confinement effects compete with the electric field to reserve the large band gap in comparison to the monolayer counterparts. In addition to decrease the band gap, the intrinsic electric field raises the VBM position at the *Γ*-point at the same time and tend to arise an indirect band gap. It can be seen that states at the *Γ*-point show substantial susceptibility to the strain and intrinsic electric filed. It thus provides us with a possibility to tune the electronic properties of TMD quantum wells by carrier doping, adjusting the strain and thickness, and applying external electric field. As demonstrated in Figure S4, the localization of VBM and CBM is also a result of the intrinsic electric field^43^,^44^.

**Figure 4:**
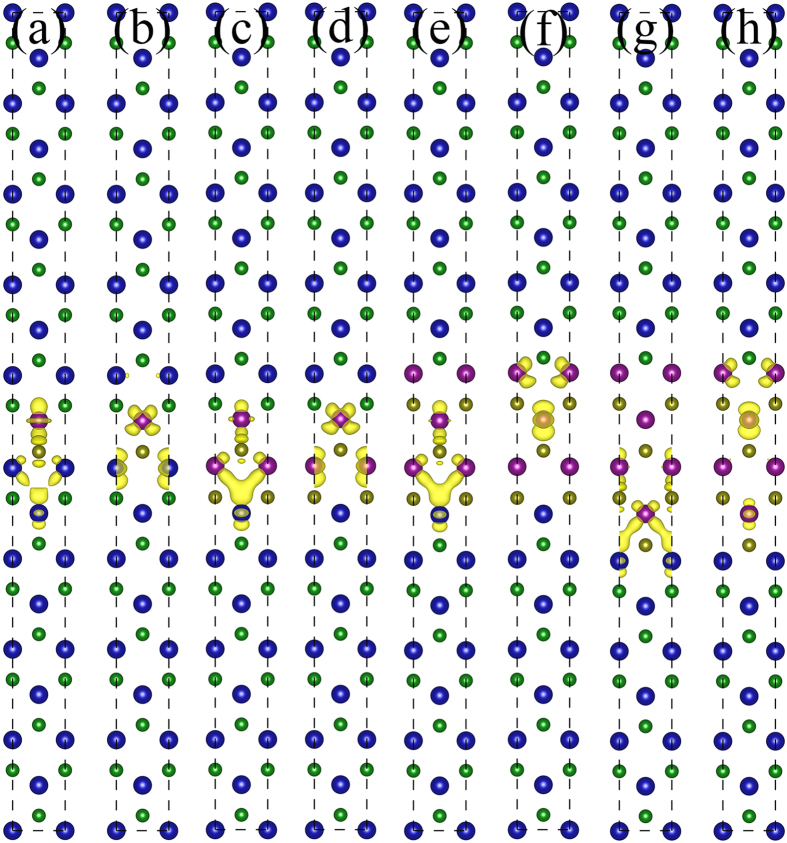
VBM and CBM at the *A*-point for WS_2_/MoSe_2_/WS_2_ quantum well with different MoSe_2_ thickness of n = 1–4. (**a,c,e,g**) represent the VBM, while (**b,d,f,h**) indicate the CBM. The small spheres are non-metal atoms (S and Se), while big spheres are metal atoms (Mo and W).

In case of WS_2_/MoSe_2_/WS_2_ quantum well, both VBM and CBM are predominantly distributed on embedded MoSe_2_, as shown in Fig. 4. As the MoSe_2_ thickness increases, the VBM and CBM tend to separate each other to locate at opposite sides of MoSe_2_ due to the intrinsic electric field, reducing the band gap. However, the separation reduces the recombination efficiency and thus affects the application in light emitting. In addition, charge transfer gives rise to band bending in constituent TMDs and strain-induced piezoelectric polarization results in quantum-confined Stark effect. As a result, further works are in need to omit these factors by, for example, carrier doping, to well confine the carriers in the wells.

**Figure 5:**
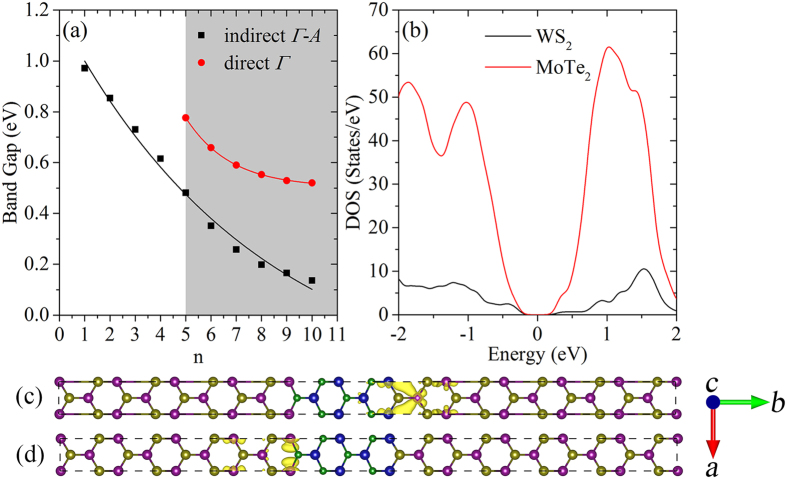
(**a**) Evolution of indirect (*Γ*-*A*) and direct (*Γ*) band gap of MoTe_2_/WS_2_/MoTe_2_ quantum wells as a function of the WS_2_ thickness n = 1–10. (**b**) DOS projected on WS_2_ and MoTe_2_ in the MoTe_2_/WS_2_/MoTe_2_ quantum well with WS_2_ thickness being n = 4; the Fermi level is set to zero. (**c**) VBM and (**d**) CBM at the *A*-point for the MoTe_2_/WS_2_/MoTe_2_ quantum well with the thickness of MoSe_2_ being n = 4. The small spheres are non-metal atoms (S and Te), while big spheres are metal atoms (Mo and W).

In MoTe_2_/WS_2_/MoTe_2_ quantum well, relatively large tensile strain is imposed on WS_2_. As discussed above, direct-indirect band gap crossover is presented due to the upward shift of VBM at the *Γ*-point as WS_2_ thickness being n = 5, as shown in Figure S5. Figure 5(a) summarizes the direct and indirect band gap values, which are smaller than 1.0 eV. The indirect band gap linearly decreases as the WS_2_ thickness increases to n = 4 and then tend to be convergent; the convergence of direct band gap is faster than that of indirect one. In comparison to WS_2_/MoSe_2_/WS_2_ quantum well, the behavior of band edge states show similar trends; however, a difference lies in the obviously smaller band gap of the MoTe_2_/WS_2_/MoTe_2_ quantum wells. In the case of n = 4, for example, the band gap of WS_2_/MoSe_2_/WS_2_ quantum well is 1.56 eV, while MoTe_2_/WS_2_/MoTe_2_ quantum well corresponds to a band gap of 0.61 eV. In WS_2_/MoSe_2_/WS_2_ quantum well, direct-indirect band gap crossover occurs with significantly larger MoSe_2_ thickness than WS_2_ due to the faster rise of VBM at the *Γ*-point in MoTe_2_/WS_2_/MoTe_2_ quantum wells. When n = 4, the density of states (DOS) plots projected on MoTe_2_ and WS_2_ are shown in Fig. 5(b), which reveals a type-I alignment with both VBM and CBM located on MoTe_2_. In contrast to the WS_2_/MoSe_2_/WS_2_ quantum well, however, the VBM and CBM are spatially separated striding the embedded WS_2_, see Fig. 5(c,d). In the presence of intrinsic electric filed, electron and hole wave functions are localized at opposite sides and such a localization is conducive to reduce the band gap. As a result, MoTe_2_/WS_2_/MoTe_2_ quantum wells manifest themselves to be promising candidates used in solar energy absorption and conversion. It is of interest and importance, fairly small band gap and physical separation of electron-hole pairs demonstrate the new possibility in achieving solar cells and photocatalysts within infrared or near infrared light region.

In GaN/InN/GaN and GaAs/Ge/GaAs quantum wells^41^,^42^, the intrinsic polarization can reduce the band gap and enhance the spin-orbital coupling (SOC) effects, driving the system to a topological insulator state. In this work, we confirm the enhanced SOC in the MoTe_2_/WS_2_/MoTe_2_ quantum well, see Figure S6, supplementary information. In TMD quantum wells, further works to realize the possibility of topological insulator states are worthy. In MoS_2_ monolayer, inversion symmetry breaking gives rise to the valley Hall effect in presence of an in-plane electric field^45^. At the end of this work, we remark that the emergent valleytronics and spintronics in TMD quantum wells are waiting for further exploration.

## Conclusion

In conclusion, we found that TMD quantum wells offer exciting new physics and hold great promise as building blocks in applications in electronics, optoelectronics as well as photocatalysts. The quantum confinement effects, strain and, in particular, intrinsic electric field collectively control the electronic structures of TMD quantum wells, which can be further controlled by artificial means to gain the desired properties. Additionally to induce large spin-orbital interaction, the sizable band gap in TMD materials can be substantially reduced and the gap nature can be controlled. It also invites further studies to realize the possibility of topological insulator states and the emergent valleytronics in such TMD quantum wells.

## Methods

The first-principles DFT calculations were performed using the projector augmented wave (PAW) scheme, as implemented in the plane-wave basis code VASP (Vienna *ab initio* simulation package)^46^,^47^. The generalized gradient approximation (GGA)^48^ as formulated by Perdew-Burke-Ernzerhof (PBE)^49^ has been introduced for exchange and correlation contributions. A cutoff energy of 400 eV was chosen for the plane-wave expansion of wave functions and the Monkhorst-Pack scheme of ***k***-point sampling has been adopted for the integration over the first Brillouin zone^50^: 13 × 13 surface grid was used to relax the two-dimensional TMD monolayers while 8 × 4 for TMD quantum wells, with the residual Hellmann-Feynman forces smaller than 0.02 eV/Å within conjugate gradient algorithm. Gaussian smearing was used to address how the partial occupancies are set for each wave function. Slab models with vacuum spacing of 18 Å was adopted to eliminate the spurious interactions between periodic imagines. Figure 1(a) shows the atomic model of TMD quantum well, in which the width of sandwiching TMD was maintained large enough in order to eliminate the effects induced by the interaction of embedded TMDs. After obtaining the optimized lattice constants of TMD monolayers, we constructed a rectangular unit cell of enough width to build quantum well structures. In the considered TMD quantum wells, on account of the experimental observation^10^,^11^,^12^,^13^,^14^, interfaces between constituent TMDs were constructed along the zigzag direction by atom substitution followed by full atomic relaxation. In this work, the lattice constants of embedded TMDs were set as that of sandwiching TMD in the calculations to mimic the laterally epitaxial growth of TMD and thus strain could be induced to the embedded TMDs^10^,^11^,^12^,^13^,^14^. According to the definition in a previous work^51^, these two-dimensional in-plane TMD A/B/A sandwich structures can be referred to as quantum wells, instead of superlattices. It should be pointed out that an interesting mechanical property of any two-dimensional layer is the existence of a huge asymmetry in mechanical instability induced by in-plane strain, which is always overlooked by many DFT calculations^52^. In our calculations, the compressive strain-induced structure undulation was not found due to the rather small size of embedded TMDs. In consideration of mechanical instability asymmetry, the electronic properties could be different; however, results for quantum wells with significantly large compressive strain induced on the embedded TMDs are helpful for well understanding the interfacing effects on the electronic structures. Although the hybrid functional or GW calculations can give band gaps more accurate, it is unrealistic to adopt for the large TMD quantum wells models considered in this work, and we confirmed that PBE results can correctly give the main features and key trends.

## Additional Information

**How to cite this article**: Wei, W. *et al.* Controlling the Electronic Structures and Properties of in-Plane Transition-Metal Dichalcogenides Quantum Wells. *Sci. Rep.* **5**, 17578; doi: 10.1038/srep17578 (2015).

## Supplementary Material

Supplementary Information

## Figures and Tables

**Table 1:** Lattice constant (*a*, in Å) of two-dimensional TMD unit cells at DFT-PBE level of theory.

TMDs	MoS_2_	MoSe_2_	MoTe_2_	WS_2_	WSe_2_	WTe_2_
*a*	3.18	3.32	3.55	3.18	3.32	3.55
