# Supplementary material for: Controlling the Electronic Structures and Properties of in-Plane Transition-Metal Dichalcogenides Quantum Wells
Source: Sci Rep. 2015 Nov 30;5:17578. doi: 10.1038/srep17578 (PMC4663467; doi:10.1038/srep17578)
Supplement: Supplementary Information [file srep17578-s1.pdf]

Supplementary Information

Controlling the Electronic Structures and Properties of in-Plane Transition-Metal Dichalcogenides Quantum Wells by Wei Wei, Ying Dai, Chengwang Niu, and Baibiao Huang

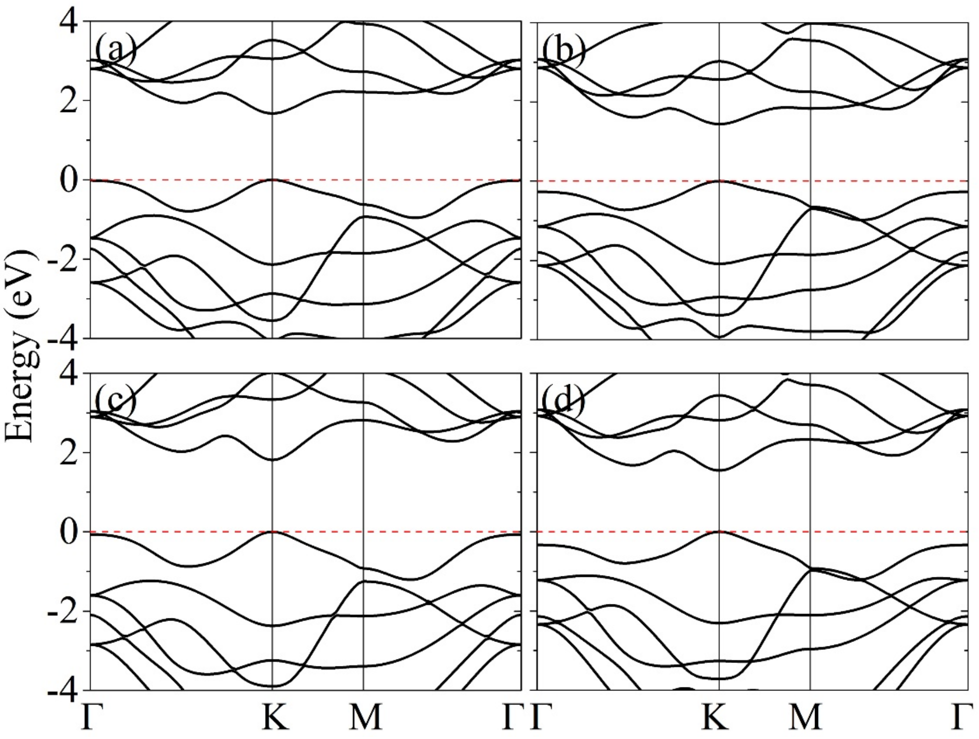

Figure S1 Band structure of two-dimensional (a) MoS_2, (b) $MoSe_2$, (c) WS_2 and (d) WSe_2. The horizontal dashed lines represent the Fermi level.

Supplementary Information

Controlling the Electronic Structures and Properties of in-Plane Transition-Metal Dichalcogenides Quantum Wells by Wei Wei, Ying Dai, Chengwang Niu, and Baibiao Huang

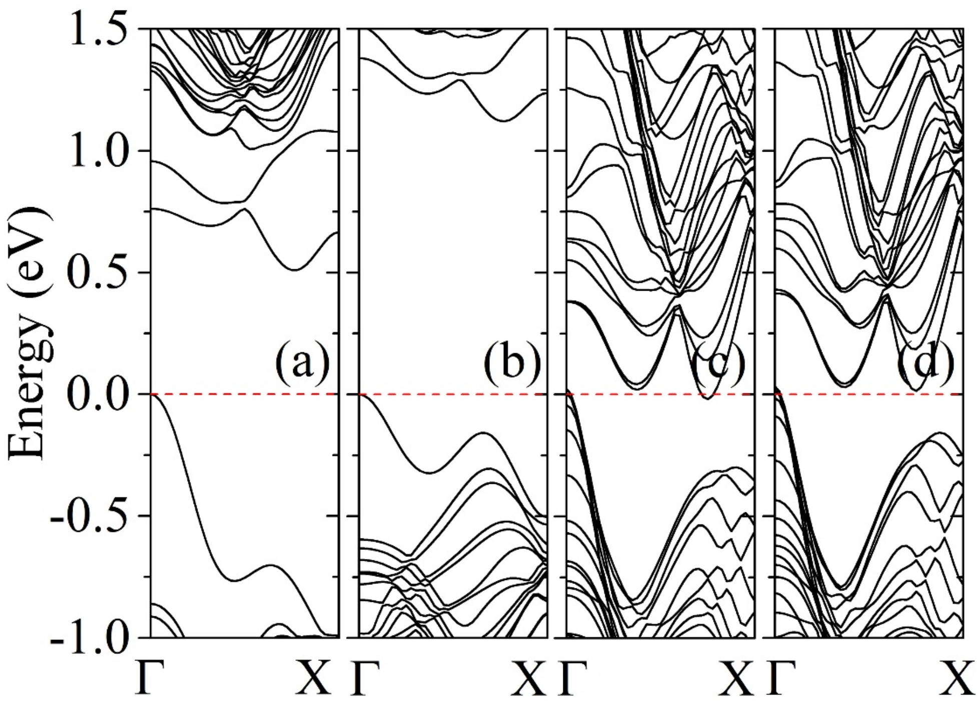

Figure S2 Band structures of TMD quantum wells with the thickness of embedded TMD being n=2 (n corresponds to the number of MX_2 units in the quantum well unit cell). (a) $WS_2/MoTe_2/WS_2$, (b) $WSe_2/MoTe_2/WSe_2$, (c) $WTe_2/MoS_2/WTe_2$, (d) $WTe_2/MoSe_2/WTe_2$. The horizontal dashed line represents the Fermi level.

Supplementary Information

Controlling the Electronic Structures and Properties of in-Plane Transition-Metal Dichalcogenides Quantum Wells by Wei Wei, Ying Dai, Chengwang Niu, and Baibiao Huang

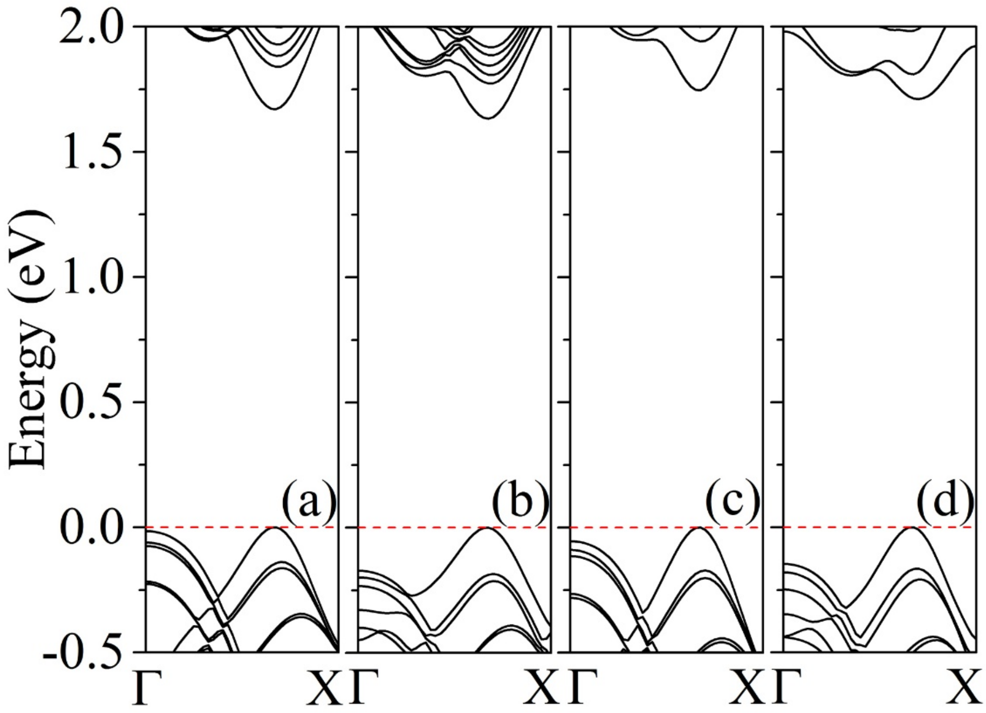

Figure S3 Band structures of TMD quantum wells with the thickness of embedded TMD being n=2 (n corresponds to the number of MX_2 units in the quantum well unit cell). (a) $MoS_2/WS_2/MoS_2$, (b) $MoS_2/WSe_2/MoS_2$, (c) $WS_2/MoS_2/WS_2$, (d) $WS_2/MoSe_2/WS_2$. The horizontal dashed lines represent the Fermi level.

Supplementary Information

Controlling the Electronic Structures and Properties of in-Plane Transition-Metal Dichalcogenides Quantum Wells by Wei Wei, Ying Dai, Chengwang Niu, and Baibiao Huang

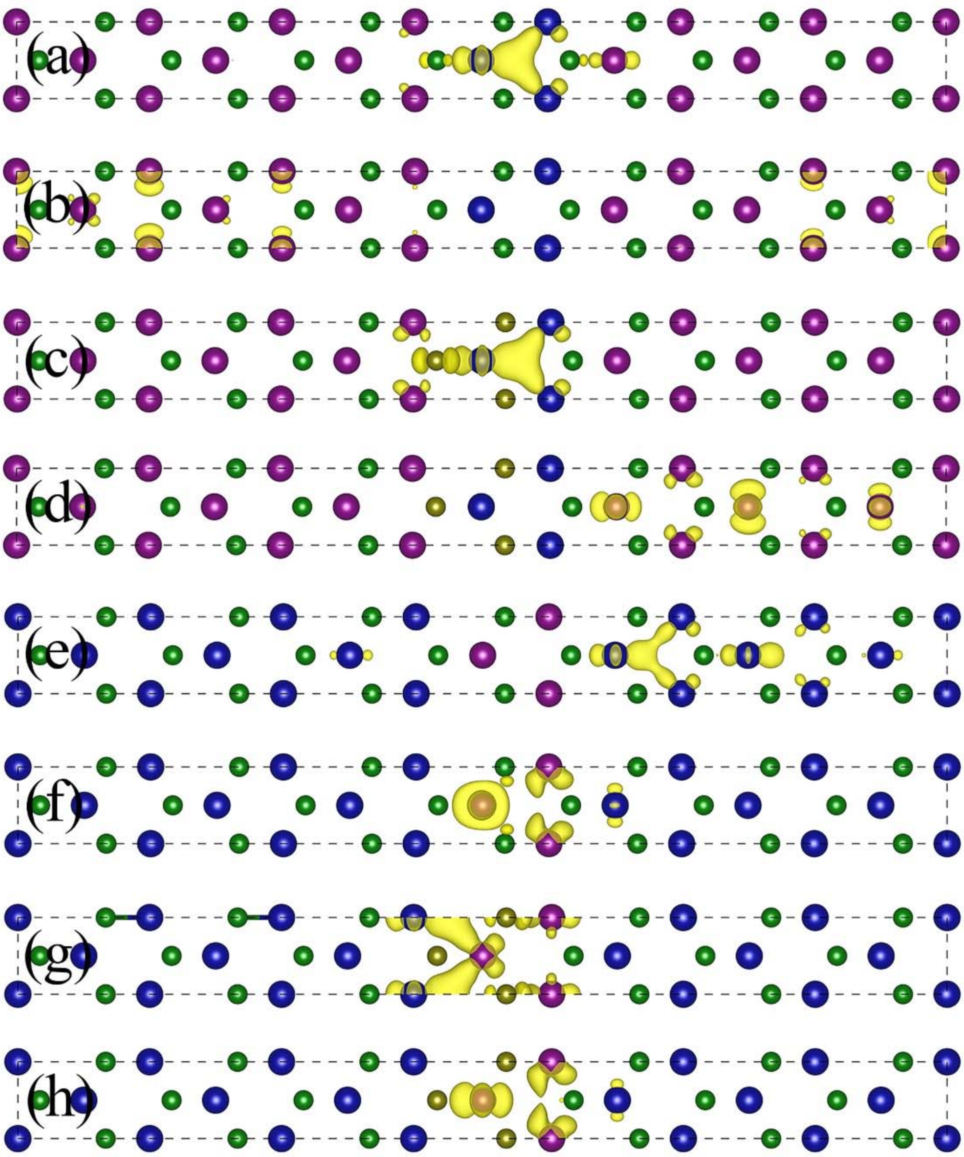

Figure S4 VBM and CBM at the *A*-point for the TMD quantum wells with the thickness of embedded TMD being n=2. VBM of (a) $MoS_2/WS_2/MoS_2$, (c) $MoS_2/WSe_2/MoS_2$, (e) $WS_2/MoS_2/WS_2$, (g) $WS_2/MoSe_2/WS_2$, and VBM of (b) $MoS_2/WS_2/MoS_2$, (d) $MoS_2/WSe_2/MoS_2$, (f) $WS_2/MoS_2/WS_2$, (h) $WS_2/MoSe_2/WS_2$. The small spheres are non-metal atoms (S and Se), while big spheres are metal atoms (Mo and W).

Supplementary Information

Controlling the Electronic Structures and Properties of in-Plane Transition-Metal Dichalcogenides Quantum Wells by Wei Wei, Ying Dai, Chengwang Niu, and Baibiao Huang

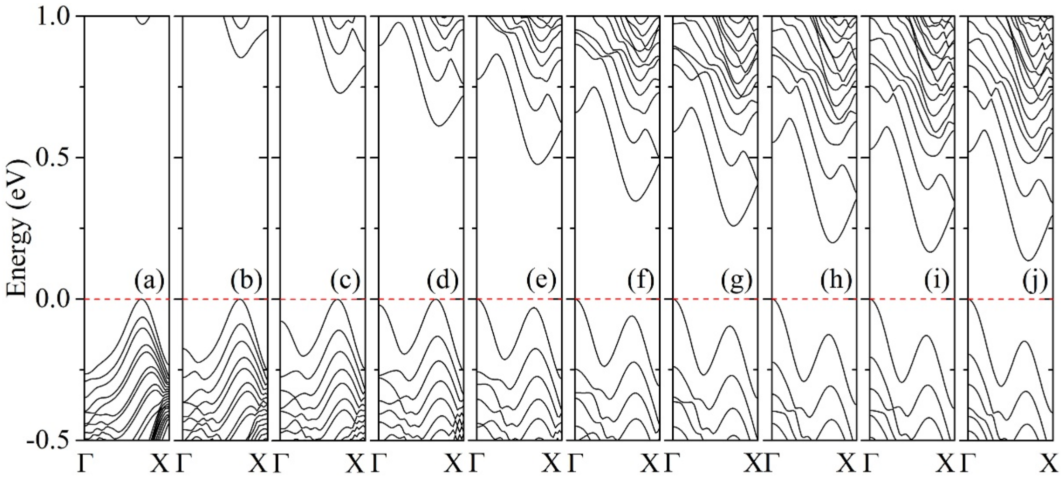

Figure S5 Band structures of $MoTe_2/WS_2/MoTe_2$ quantum well with the thickness of WS_2 being n=1-10 (n corresponds to the number of WS_2 units in the $MoTe_2/WS_2/MoTe_2$ quantum well unit cell). The horizontal dashed lines represent the Fermi level.

Supplementary Information

Controlling the Electronic Structures and Properties of in-Plane Transition-Metal Dichalcogenides Quantum Wells by Wei Wei, Ying Dai, Chengwang Niu, and Baibiao Huang

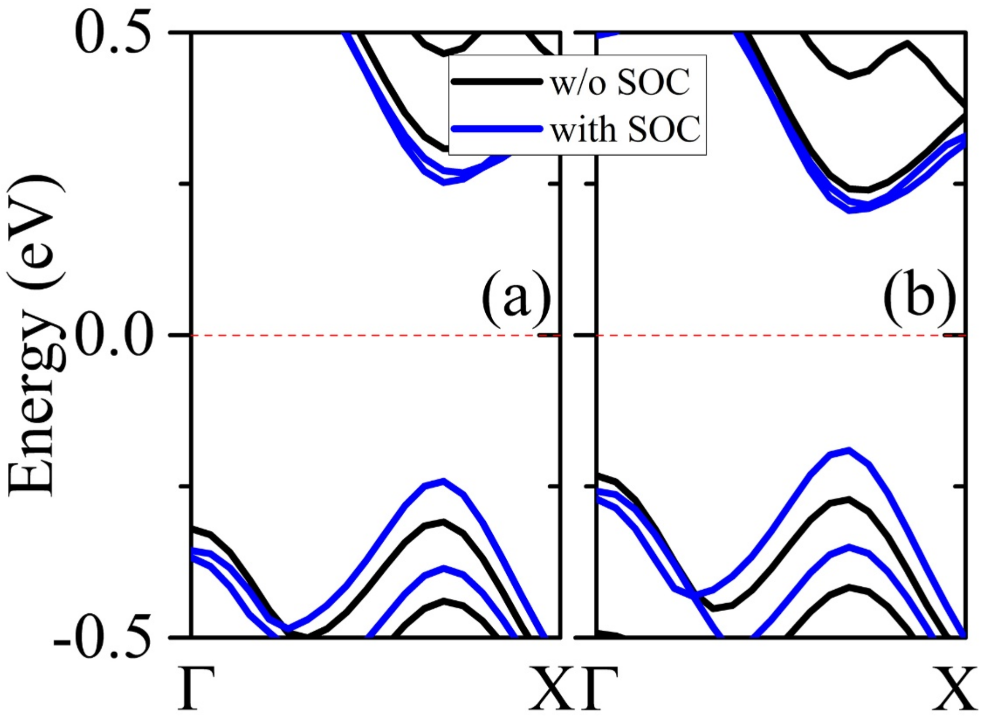

Figure S6 Band structures of $MoTe_2/WS_2/MoTe_2$ quantum well with the thickness of WS_2 being (a) n=4 and (b) n=5 (n corresponds to the number of WS_2 units in the $MoTe_2/WS_2/MoTe_2$ quantum well unit cell) with and without (w/o) the consideration of SOC. The horizontal dashed lines represent the Fermi level.
